# Pseudo-Thrombotic Thrombocytopenic Purpura Due to Severe Vitamin B12 Deficiency: A Case Report

**DOI:** 10.7759/cureus.40212

**Published:** 2023-06-10

**Authors:** Thanmay Sathi, Kunjal Luhadia, Kanica Yashi, Jaswinder Virk, Taral Parikh, Megha Dogra, Ahmad S Alam

**Affiliations:** 1 Internal Medicine, Bassett Healthcare, Cooperstown, USA; 2 Cardiology, Bassett Healthcare, Cooperstown, USA; 3 Pediatrics, Hamilton Health Center, Harrisburg, USA; 4 Internal Medicine, Saint Vincent Hospital, Worcester, USA; 5 General Surgery, John Hunter Hospital, Newcastle, AUS

**Keywords:** vit b12 deficiency, pseudo-thrombotic thrombocytopenic purpura, auto immune, hemolytic uremic syndrome (hus), acquired ttp, pseudo-ttp, vitamin b deficiency

## Abstract

Vitamin B12 deficiency is common in vegetarians, as meat is a common source of vitamin B12. In this case presentation, a patient presented to his primary care doctor with signs of severe vitamin B12 deficiency anemia. He had elevated lactate dehydrogenase levels, indirect bilirubin, and schistocytes on the blood smear, all pointing toward a hemolytic process. A severe vitamin B12 deficiency was deemed the cause of this hemolytic anemia after ruling out other causes. We highlight the importance of knowing more about this pathogenesis to avoid unnecessary workup and management for an elementary disorder that can result from severe B12 deficiency.

## Introduction

Pseudo-thrombotic microangiopathy is a condition that is often mistaken for thrombotic thrombocytopenic purpura (TTP) or hemolytic uremic syndrome (HUS). It is an uncommon clinical presentation known to occur in 2.5% of cases of cyanocobalamin deficiency, commonly known as vitamin B12 deficiency. It presents clinical findings of anemia, thrombocytopenia, and schistocytosis in severe vitamin B12 deficiency. Primary thrombotic microangiopathy (TMA) describes a specific pathologic lesion involving the vessel walls of arterioles and capillaries, leading to microvascular thrombosis. Primary TMA includes thrombotic thrombocytopenic purpura, Shiga toxin-mediated hemolytic uremic syndrome, drug-induced TMA, and complement-mediated TMA. The hallmark of diagnosis is microangiopathic hemolytic anemia, thrombocytopenia, renal and neurological abnormalities, and gastrointestinal and cardiac features [1, 2].

Vitamin B12 is absorbed into the body through the ileum as part of the vitamin B12-intrinsic factor complex. Physiologically, vitamin B12 plays a vital role, along with folate, in deoxyribonucleic acid (DNA) and ribonucleic acid (RNA) synthesis. Vitamin B12 helps in the enzymatic breakdown of folate into dihydrofolate and, subsequently, tetrahydrofolate. This chemical reaction is coupled with the conversion of homocysteine to methionine. Defective folate reduction leads to defective DNA synthesis in the S phase of the cell cycle, which leads to defective nuclear maturation in the setting of normal cytoplasmic maturation, leading to megaloblastic changes in the bone marrow [3, 4].

The pathophysiology of pseudo-thrombosis is poorly understood, but there are two proposed mechanisms: intramedullary hemolysis and hyperhomocysteinemia. Intramedullary hemolysis occurs due to fragile red blood cell (RBC) membranes, which causes shearing before the cells reach the peripheral circulation. Elevated homocysteine levels lead to the accumulation of reactive oxygen species, which damage the endothelium and further lyse the fragile RBCs [5-8].

It is essential to distinguish pseudo-thrombotic microangiopathy from TTP and HUS to avoid expensive treatments like plasmapheresis, which has a spectrum of adverse effects, including electrolyte abnormalities, coagulation factor abnormalities, allergic reactions, and problems with fluid overload. It is also necessary to prevent unnecessary plasma exchange treatments due to the high treatment costs and burden on the health care system and society incurred by these treatments [4-6].

## Case presentation

A 69-year-old man `with a history of gastroesophageal reflux disease (GERD) presented to his primary care physician for an acute visit for a new-onset rash in the inguinal area (for the past seven days), marked fatigue, and 10 pounds of weight loss (over the past few months). He also reported a decreased appetite and had symptoms of GERD, for which he was started on famotidine but was not compliant. On presentation, his blood pressure was 102/58, his pulse was 100, his temperature was 36.9°C (98.4°F), and his respiratory rate was 12 breaths per minute (Table 1).

**Table 1:** The patient's vital signs on presentation

Vital signs	
Blood pressure	102/58
Temperature	36.9 °C (98.4 °F)
Respiratory rate (breaths per minute)	12
Pulse rate	100

Cardiovascular, respiratory, neurologic, and abdominal exams were unremarkable. The patient had marked erythema in the left inguinal region and some scant purulent discharge from the skin folds.

He had been prescribed cephalexin and topical ketoconazole for the rash, and his initial lab workup showed a hemoglobin drop from 14 to 7.5 g/dL, a mean corpuscular volume (MCV) of 124, and a vitamin B12 level of less than 50. For this reason, hospitalization was recommended for further evaluation and treatment.

At the presentation to the emergency room for further history, it was found that he had no melena, hematemesis, blood in the sputum, or any other bleeding site. A fecal occult blood test (FOBT) was negative. The last colonoscopy done in 2009 did not show any abnormal growth.

Labs on the following day of the hospitalization indicated lactate dehydrogenase (LDH) of 824 U/L. A complete blood count (CBC) revealed thrombocytopenia of 109 and 1+ schistocytes. His total bilirubin was 2.1, and indirect bilirubin was 1.6; folate and thyroid-stimulating hormone (TSH) were normal.

As for management, he received two units of blood transfusions and vitamin B12 1000mcg injections daily. At this point, hematology was consulted due to a concern about drug-induced hemolytic anemia, and they recommended starting treatment with high-dose prednisone followed by a taper. A haptoglobin and direct antiglobulin test (DAT) were obtained, based on which a decision to stop steroids would be made if the tests returned negative. The DAT returned negative, ruling out drug-induced hemolytic anemia; however, the steroids continued since the patient was on a steroid taper.

On follow-up with hematology after a month, it was found that his haptoglobin was less than 15. His repeat bloodwork showed normalization of all his lab values, including hemoglobin, MCV, bilirubin, and lactate dehydrogenase.

## Discussion

The patient’s lab values improved after vitamin B12 supplementation, and his Coombs test was negative. His symptoms of anemia were present before he was prescribed cephalexin (it is unsure if he even started taking the medication), so even though he received a steroid taper, we think the hemolytic anemia was due to the severe vitamin B12 deficiency. There have been other case reports that have had a similar presentation [1].

Vitamin B12 deficiency is common in the United States. According to the National Institutes of Health (NIH), 6% of adults younger than 60 in the United States and the United Kingdom have a vitamin B12 deficiency. The rate is closer to 20% for people over 60. The significant causes of deficiency include difficulty absorbing vitamin B12 from food, lack of intrinsic factor (pernicious anemia), gastrointestinal tract surgery, prolonged use of certain medications (proton pump inhibitors (PPIs)), and dietary deficiency. The most common causes of deficiency are pernicious anemia and dietary causes. The NIH estimates that 10% of vitamin B12 deficiency cases present with pancytopenia or hemolysis. In a case series of 201 patients, it was estimated that 2.5% of patients with vitamin B12 deficiency exhibited pseudo-thrombotic microangiopathy. It is estimated that around 38.8% of the cases of pseudo-thrombotic microangiopathy are misdiagnosed as thrombotic thrombocytopenic purpura and receive plasma exchange therapy [3].

The pathogenesis of pseudo-TMA is thought to be intramedullary hemolysis, leading to peripheral pancytopenia. The immature erythrocytes, being extremely fragile, are sheared easily, producing schistocytes. This happens in the absence of platelet microthrombi. The clinical picture is similar to TTP, with the difference being reticulocytopenia, considered a universal finding in cases of pseudo-thrombotic microangiopathy. The mechanism is thought to be insufficient vitamin B12 for sufficient compensatory erythropoiesis. Another mechanism is thought to be hyperhomocysteinemia, as it is not biochemically converted to methionine without cyanocobalamin. Homocysteine exerts a cytotoxic effect because of its pro-oxidant nature. Homocysteine also down-regulates glutathione peroxidase activity, leading to the intracellular accumulation of reactive oxygen species and the destruction of erythrocytes. Also, it is thought that oxidative stress damages the endothelial cells, leading to microangiopathy and platelet microthrombi, causing shearing of the erythrocytes and subsequent schistocyte formation [2].

It is crucial to differentiate pseudo-TMA from actual TMA for multiple reasons. The primary reason is the simplicity of treating vitamin B12 deficiency and preventing additional healthcare costs and burdens. For instance, TTP is an emergency and would require urgent plasmapheresis, which is not readily available at all centers, and so for this reason, the patient would have to be transferred out [4, 6].

Furthermore, as we know, vitamin B12 deficiency can be easily prevented by knowing more about the side effects; primary care providers and even internists at the hospital can be more cautious with patients specifically prone to developing this. For instance, patients who are vegetarians or others with malabsorption tendencies, such as bariatric surgery patients [9-12].

## Conclusions

We want to show through this case report that pseudo-TMA is a relatively benign medical condition that can be easily treated. However, clinically, it mimics TTP, which is a medical emergency that needs to be promptly treated. As it closely resembles TTP, many clinicians treat it as TTP and start the patients on plasmapheresis, which has its own complications and also increases the burden on the healthcare system.

A patient with TTP typically presents with a clinical spectrum of fever, microangiopathic hemolytic anemia, thrombocytopenia, renal abnormalities, and neurological abnormalities. If the patient does not have too many of the mentioned clinical signs and symptoms, it is advisable to screen for other causes. Obtaining blood for testing vitamin B12 levels can quickly identify vitamin B12 deficiency, and supplementation can be done promptly.
